# Retraction: ‘Long non-coding RNA LINC00152 promotes gallbladder cancer metastasis and epithelial-mesenchymal transition by regulating HIF-1α via miR-138’

**DOI:** 10.1098/rsob.230036

**Published:** 2023-02-10

**Authors:** Qiang Cai, Zhenqiang Wang, Shouhua Wang, Mingzhe Weng, Di Zhou, Chen Li, Jiandong Wang, Erzhen Chen, Zhiwei Quan

*Open Biol.* **10**, 20170247. (Published online 1 January 2017) (https://doi.org/10.1098/rsob.160247)

Following an investigation, we have found that in figures 4, 5 and 6 of the manuscript, there appears to be multiple duplications, re-positioning and alterations to the western blot images. In further review of the images and the raw data supplied by the authors, we are unable to verify the conclusions of the paper or validity of the data.

The image integrity standards and policies for the journal can be found here: https://royalsocietypublishing.org/doi/10.1098/rsob.200165.

Prof. Jon Pines FRS Editor-in-Chief, *Open Biology*

*Open Biology* Editorial Team

